# Carotid Atherosclerotic Calcification Characteristics Relate to Post-stroke Cognitive Impairment

**DOI:** 10.3389/fnagi.2021.682908

**Published:** 2021-05-25

**Authors:** Yingzhe Wang, Chanchan Li, Mengyuan Ding, Luyi Lin, Peixi Li, Yizhe Wang, Qiang Dong, Yanmei Yang, Mei Cui

**Affiliations:** ^1^Department of Neurology, Huashan Hospital, Fudan University, Shanghai, China; ^2^Department of Radiology, Huashan Hospital, Fudan University, Shanghai, China; ^3^Department of Medicine, Nanchang University, Nanchang, China; ^4^The State Key Laboratory of Medical Neurobiology, Fudan University, Shanghai, China

**Keywords:** stroke, cognition, atherosclerosis, calcification, Computed Tomographic Angiography

## Abstract

**Background:** Together with cerebral small vessel disease (CSVD), large vessel atherosclerosis is considered to be an equally important risk factor in the progression of vascular cognitive impairment. This article aims to investigate whether carotid atherosclerotic calcification is associated with the increased risk of post-stroke cognitive impairment (PSCI).

**Methods:** A total of 128 patients (mean age: 62.1 ± 12.2 years, 37 women) suffering from ischemic stroke underwent brain/neck computer tomography angiography examination. The presence and characteristic of carotid calcification (size, number and location) were analyzed on computer tomography angiography. White matter hyperintensity (WMH) was assessed using Fazekas scales. PSCI was diagnosed based on a battery of neuropsychological assessments implemented 6−12 months after stroke.

**Results:** Among 128 patients, 26 developed post-stroke dementia and 96 had carotid calcification. Logistic regression found carotid calcification (odds ratio [OR] = 7.15, 95% confidence interval [CI]: 1.07–47.69) and carotid artery stenosis (*OR* = 6.42, 95% CI: 1.03–40.15) both significantly increased the risk for post-stroke dementia. Moreover, multiple, thick/mixed, and surface calcifications exhibited an increasing trend in PSCI (*P*_*trend*_ = 0.004, 0.016, 0.045, respectively). The prediction model for post-stroke dementia including carotid calcification (area under curve = 0.67), WMH (area under curve = 0.67) and other covariates yielded an area under curve (AUC) of 0.90 (95% CI: 0.82–0.99).

**Conclusion:** Our findings demonstrated that the quantity and location of carotid calcifications were independent indicators for PSCI. The significant role of large vessel atherosclerosis in PSCI should be concerned in future study.

## Introduction

Stroke is considered to be one of the most widespread and serious cerebrovascular diseases affecting millions of people worldwide. With a high prevalence of 20–80%, post-stroke cognitive impairment (PSCI) is one of the major complications suffered during the chronic stage of ischemic stroke (Sun et al., 2014). As cognitive function can fluctuate due to neurological deficits and subsequent improvement of perfusion in early phases, the diagnosis of PSCI is often postponed by at least 3 months after the onset of stroke (Gottesman and Hillis, 2010). Thus, early identification of patients at high-risk of PSCI, based on patients’ baseline characteristics, is essential in the orchestration of appropriate preventataive management.

Several factors, including age, education level, vascular risk factors, extent of stroke, and neuroimaging features, are considered to be important determinants of PSCI (Rasquin et al., 2004; Leys et al., 2005; Lu et al., 2016). Apart from acute stroke, pre-existing cerebral small vessel disease (CSVD) is believed to be closely connected with both the prognosis of stroke and the occurrence of cognitive dysfunction. It has been reported that PSCI was significantly associated with several subtypes of baseline CSVD, including white matter hyperintensity (WMH), cerebral microbleed, enlarged perivascular space and brain atrophy (Wen et al., 2004; Gregoire et al., 2012; Kebets et al., 2015; Molad et al., 2017; Arba et al., 2018). Interestingly, although CSVD is often accompanied by pathological changes in large arteries, the relationship between large vessel diseases (such as stenosis, slow blood flow, malformation, or poor collateral circulation) and PSCI remains ambiguous. While some research has demonstrated that large vessel stenosis was highly predictive of PSCI (Kandiah et al., 2016; Li et al., 2017), not all studies have shown consistent results (Chaudhari et al., 2014). Understanding the relationship between atherosclerotic calcification and PSCI will provide a deeper insight into the pathology of PSCI, which would be beneficial for early diagnosis and prevention.

Therefore, in this study, we aimed to investigate whether the presence of carotid atherosclerotic calcification is an associated risk factor for the development and progression of PSCI, and whether this association depends on the characteristic of calcification.

## Materials and Methods

### Study Population and Design

We utilized a dataset from a prospective study conducted in the Stroke Unit of Huashan Hospital, Shanghai. This longitudinal cohort was designed to investigate the risk factors, clinical features and outcomes of PSCI. The inclusion criteria were as follows: (a) diagnosed with ischemic stroke within 7 days of onset; (b) age ≥ 18 years; (c) National Institute of Health stroke scale (NIHSS) score ≤ 25; (d) able and willing to accept brain imaging examination and neuropsychological assessment. Subjects with following conditions were excluded: (a) pre-stroke dementia; (b) unable to speak or write due to aphasia or paralysis; (c) renal dysfunction; (d) allergy to iodine; (e) contraindications to magnetic resonance imaging (MRI) examination;(f) patients with abnormal calcium and phosphorus metabolism; (g) known nervous system disease or severe chronic medical disease; (h) mental health conditions, such as anxiety or depression; (i) pregnancy.

The study was approved by the Ethics Committee of Fudan University, Shanghai, China. All participants signed written informed consent before data collection.

### Imaging Acquisition

All patients underwent cranio-cervical computer tomography angiography (CTA) within 7 days of stroke onset. CTA was performed on a 256-section scanner (Brilliance i CT, Phillips Medical Systems, Ohio, United States) from the aortic arch to the vertex with parameters as follows: 120 kVp, 150 mAs, slice thickness of 1 mm, pitch of 0.7, field of view of 220 mm, matrix of 512 × 512, helical scanning mode and intravenous administration of 50 mL non-ionic contrast (Ultravist, Bayer Healthcare, Berlin, Germany) at 5 mL/s via power injector (Stellate Injection System, Indianola, PA, United States) with an 8 s delay. CTA raw data were processed using the Phillips Brilliance Workspace portal software (Vision 5.0.2), including multi-planar reformation, curved planar reformation, maximum intensity projection and volume rendering. All MRI examinations were performed on a 3.0 T scanner (GE Discovery750, Milwaukee, United States, or Siemens MAGNETOM Verio, Erlangen, Germany) using a pre-programmed protocol. The main MRI sequences included: T1-weighted, T2-weighted fluid attenuated inversion recovery, diffusion-weighted imaging, and apparent diffusion coefficient.

### Imaging Interpretation

All CTA and MRI images were reviewed and analyzed by two independent neuroradiologists (C.C. Li and L.Y. Lin) with over 3 years’ experience in neurovascular imaging. Inconsistent cases were handed to another senior neuroradiologist (Y.M. Yang) and the final decision was made based on their consensus. Raters were blinded to all clinical data.

**FIGURE 1:**
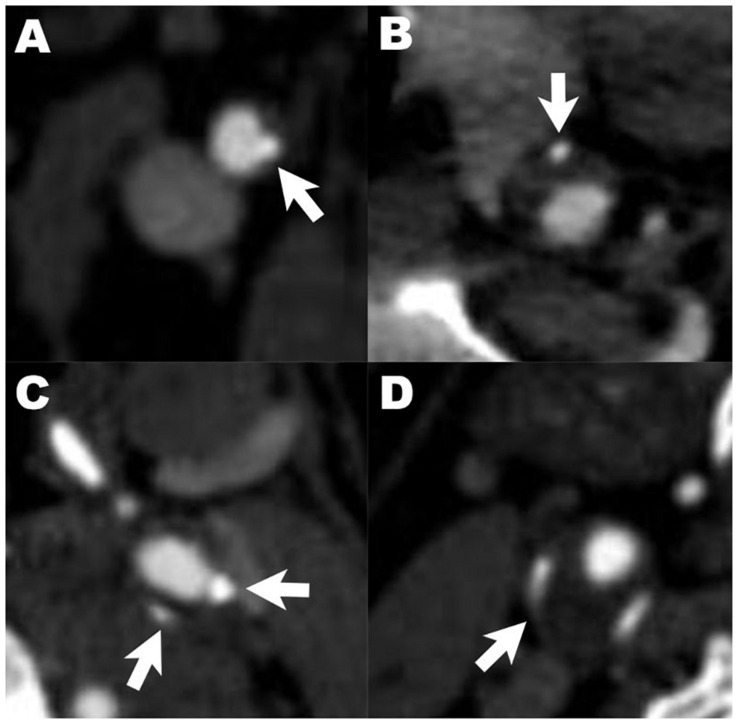
Examples of different radiological characteristics of calcified plaques. Arrows indicate calcification with different characteristics: **(A–C)** describe the relative location of calcified plaques in carotid artery **(A)**, surface calcification; **(B)**, deep calcification; **(C)**, mixed calcification). Panel **(D)** shows calcification with positive rim sign.

Carotid arteries were divided into three segments: common carotid arteries, cervical internal carotid arteries, and intracranial internal carotid arteries. The presence, location, number and maximum thickness of calcification on unilateral or bilateral carotid arteries were analyzed and recorded. All arteries with calcified plaques were annotated as single (<2) or multiple (≥2), according to the number of plaques. The calcification location was categorized as surface, deep or mixed (with both surface and deep calcification). Surface calcification was defined as a calcified nodule located within or close to the intimal lumen interface. Deep calcification was defined as a calcified nodule located within or close to the media/adventitia border, with fibrous tissue completely covering (Lin et al., 2017). Calcification was also classified as thin (<2 mm), thick (≥2 mm) or mixed (with both thin or thick calcification) based on the maximum thickness (Yang et al., 2018). Rim sign was defined as thin calcification (<2 mm) within the adventitia with thick internal soft plaque (≥2 mm) inside (Eisenmenger et al., 2016). Examples of different radiological characteristic of calcification are illustrated in Figure 1.

The degree of carotid artery stenosis, intracranial artery stenosis (ICAS), remodeling index (RI), and soft plaque density was further analyzed. Degree of lumen stenosis was calculated and divided into three groups: mild (0−49.9%), moderate (50−69.9%), and severe (70−99.9%), following the criteria of the North American Symptomatic Carotid Endarterectomy Trial (Committee, 1991). No patients with carotid artery occlusion were enrolled in this study. Intracranial large arteries were categorized into anterior (including anterior cerebral artery, internal carotid artery, and middle cerebral artery) and posterior (including posterior cerebral artery, vertebral artery, and basal artery) circulations. ICAS was defined as moderate–severe stenosis (≥50%) of each artery following the criteria of Warfarin-Aspirin Symptomatic Intracranial Disease (Samuels et al., 2000). RI was defined as the ratio of the cross-sectional vessel area at the maximal stenotic site to the reference vessel area at the nearby disease-free site (Miura et al., 2011). Soft plaque density was analyzed using the mean Hounsfield unit across the whole plaque volume (U-King-Im et al., 2010).

Lacune and WMH were diagnosed according to the Standards for Reporting Vascular Changes on Neuroimaging criteria (Wardlaw et al., 2013). WMH was categorized into three groups based on the sum of Fazekas scales (periventricular and deep): mild(0−2), moderate(3−4), severe(5−6).

### Cognitive Function Assessment

Patients’ cognitive function was assessed within 7 days of admission and at 6–12 months using the neuropsychological battery, which included global function and separate cognitive domains: (1) Global function: Mini–mental State Examination (MMSE) and Montreal Cognitive Assessment; (2) Memory and execution: memory and executive screening scale; (3) Visuospatial function: visuospatial overlapping diagram from Montreal Cognitive Assessment−Basic; and (4) Language: language screening test. The cut-off was determined by mean − 1.0 standard deviations (SD) or standardized values for all the tests. A patient was considered having PSCI if at least one cognitive domain (memory, execution, visuospatial function and language) was impaired (Jacquin et al., 2014). Among patients with PSCI, post-stroke dementia (PSD) was defined using interviews, neuropsychological battery and clinical dementia rating in accordance with the *Diagnostic and Statistical Manual of Mental Disorders, Fourth Edition*. Those who did not meet the criteria of PSD were classified as post-stroke cognitive impairment with no dementia (PSCIND). Pre-stroke dementia, anxiety and depression was assessed and excluded using informant questionnaire on cognitive decline in the elderly, geriatric depression scale and the 12-item neuropsychiatric inventory questionnaire, separately. All neuropsychological assessments were administered by experienced neurologists (M.Y. Ding, Y.Z. Wang and M. Cui) in our team.

### Covariates

Demographic and clinical characteristic data was collected from the database of stroke patients at baseline and follow-up. Obtained information included demographics (age, sex, years of education, body mass index, etc.), vascular risk factors (hypertension, diabetes mellitus, hyperlipidemia, etc.), lifestyle (smoking, alcohol consumption, etc.), stroke severity (NIHSS score), and CSVD (lacune, WMH). Hypertension was defined as systolic pressure ≥140 mmHg and/or diastolic pressure ≥90 mmHg, or the patient having been previously diagnosed and treated. Diabetes mellitus was defined as fasting plasma glucose ≥7.0 mmol/L, or postprandial 2 h glucose ≥11.1 mmol/L, or the patient having been previously diagnosed and treated. Hyperlipidemia was defined as total cholesterol ≥5.2 mmol/L, or low-density lipoprotein cholesterol ≥2.6 mmol/L, or triglycerides ≥1.70 mmol/L, or the patient having been previously diagnosed and treated. Smoking and alcohol consumption were divided into two statuses: current (at least one cigarette per 3 days or at least 50 grams of alcohol per week in the past 6 months) and past (quit smoking or drinking for at least 1 year) (Li et al., 2019).

### Statistical Analyses

Variable normality was determined using Kolmogorov−Smirnov test. Continuous variables are presented as mean ± standard deviation (in normality distribution) or median with interquartile range (IQR) (in skewness distribution) and compared using Student’s *t*-test or Mann−Whitney *U* test. Categorical variables are displayed as count with frequency (%) and compared using Pearson’s chi–square test or rank sum test (for ordinal categorical variables). Multinomial logistic regression was conducted to estimate the odds ratios (OR), 95% confidence intervals (CI) of PSCIND and PSD based on carotid plaque characteristics. Binary logistic regression with the selected covariates was used to calculate the area under the receiver operator characteristic curve for predicting PSD. Linear regression was performed to investigate the association of carotid atherosclerosis and post-stroke cognitive function in different domains. The scores of each cognitive test were transformed into *Z*-scores [(individual test score – mean score)/SD]. Ordinal logistic regression was performed to obtain *P*-trend value by treating post-stroke cognitive function as an ordinal variable.

Demographics, vascular risk factors and stroke severity were adjusted as covariates in regression models, including age, sex, years of education, NIHSS score at baseline (model 1), as well as hypertension, hyperlipidemia, WMH, and carotid artery stenosis (model 2). All statistical analyses were performed using SPSS v19.0 or R v3.6.2 for Mac OS. *P* < 0.05 was considered to be statistically significant.

## Results

One hundred seventy-nine participants who met the criteria from June 2017 to May 2018 were included in this study. Each individual was given a standardized treatment scheme during hospitalization. The median follow-up time was 6.9 months for this study population. Among them, 20 subjects did not undergo brain or neck CTA at baseline. Of the remaining 159 individuals, 29 refused to accept neuropsychological assessment at follow-up; 2 were excluded due to the recurrence of stroke. Therefore, a total of 128 individuals with complete clinical information were included in the final analysis. The flow chart of the enrollment procedures of study individuals is shown in Supplementary Figure 1. No significant difference between the baseline profiles of participants involved in this study and those lost in follow-up was found (Supplementary Table 1).

### Basic Characteristics

The basic characteristics of the study population are demonstrated in Supplementary Table 1. The mean age (SD) was 62.1 (12.2) years and 37 (28.9%) of the subjects were women. The population had a median (IQR) 12.0 (9.0, 15.0) years of education. Median (IQR) NIHSS score at baseline was relatively low, at 4.0 (2.0, 6.0). Lacune and severe WMH were found in 53 (41.4%) and 21 (16.4%) patients, respectively. Among all individuals, 96 subjects (75%) had calcification on unilateral or bilateral carotid arteries. 21 subjects (16.4%) had moderate to severe carotid artery stenosis. Cognitive function assessment classified individuals as having normal cognition (*N* = 27), PSCIND (*N* = 75), or PSD (*N* = 26) (Table 1). Compared to participants with normal cognition, those who have PSCI (both PSCIND and PSD) were older, less educated, scored higher on NIHSS and grading of WMH at baseline using univariate analyses (*P* < 0.05). The PSCI group also had a higher proportion of calcification and moderate or severe carotid artery stenosis (only in PSD group) (*P* < 0.05). There was no significant association between soft plaque density, RI, ICAS, other vascular risk factors and PSCI.

**TABLE 1:** Comparison of clinical and imaging characteristics between groups with different post-stroke cognitive function.

	**Normal (*N* = 27)**	**PSCIND (*N* = 75)**	**PSD (*N* = 26)**	***P*-value (PSCIND vs. Normal)**	***P-*value (PSD vs. Normal)**
**Demographic characteristics**					
Age, mean ± SD	52.6 ± 15.6	64.3 ± 10.0	65.3 ± 9.0	0.001	0.001
Female, n (%)	7 (25.9)	19 (25.3)	11 (42.3)	0.952	0.208
Education year, median (IQR)	12.0 (9.8, 15.8)	12.0 (9.0, 12.0)	9.0 (3.0, 11.0)	0.017	0.001
Smoking (ever), n (%)	14 (51.9)	36 (48.0)	15 (57.7)	0.731	0.669
Alcohol consumption (ever), n (%)	7 (25.9)	28 (37.3)	7 (26.9)	0.284	0.934
Hypertension, n (%)	16 (59.3)	57 (76.0)	20 (76.9)	0.098	0.168
Diabetes mellitus, n (%)	5 (18.5)	23 (30.7)	10 (38.5)	0.225	0.107
Hyperlipidemia, n (%)	11 (40.7)	31 (41.3)	9 (34.6)	0.957	0.646
**Baseline stroke severity**					
NIHSS at baseline, median (IQR)	2.0 (1.0, 4.5)	4.0 (2.0, 6.0)	5.0 (3.0, 8.0)	0.003	0.001
**Cerebral small vessel diseases**					
Lacune, n (%)	10 (37.0)	30 (40.0)	13 (50.0)	0.576	0.590
WMH					
Mild, n (%)	13 (48.1)	13 (17.3)	1 (3.8)	0.006	0.001
Moderate, n (%)	10 (37.0)	49 (65.3)	21 (80.8)		
Severe, n (%)	4 (14.8)	13 (17.3)	4 (15.4)		
**Atherosclerotic characteristics**					
Calcification, n (%)	14 (51.9)	59 (78.7)	23 (88.5)	0.008	0.004
Soft plaque density (Hu), mean ± SD	38.8 ± 32.2	36.2 ± 19.7	47.4 ± 27.7	0.854	0.553
Remodeling index, median (IQR)	1.0 (1.0, 1.2)	1.0 (1.0, 1.2)	1.0 (1.0, 1.4)	0.290	0.870
Carotid artery stenosis					
Mild, n (%)	26 (96.3)	63 (84.0)	18 (69.2)	0.222	0.024
Moderate or severe, n (%)	1 (3.7)	12 (16.0)	8 (30.8)		
Intracranial artery stenosis					
Anterior arteries, n (%)	7 (25.9)	25 (33.3)	14 (53.8)	0.601	0.135
Posterior arteries, n (%)	4 (14.8)	15 (20.0)	6 (23.1)	0.505	0.669

*IQR, interquartile range; NIHSS, National Institute of Health stroke scale; PSCIND, post-stroke cognitive impairment non-dementia; PSD, post-stroke dementia; WMH, white matter hyperintensity.*

### Carotid Calcification and Post-stroke Cognitive Impairment

**FIGURE 2:**
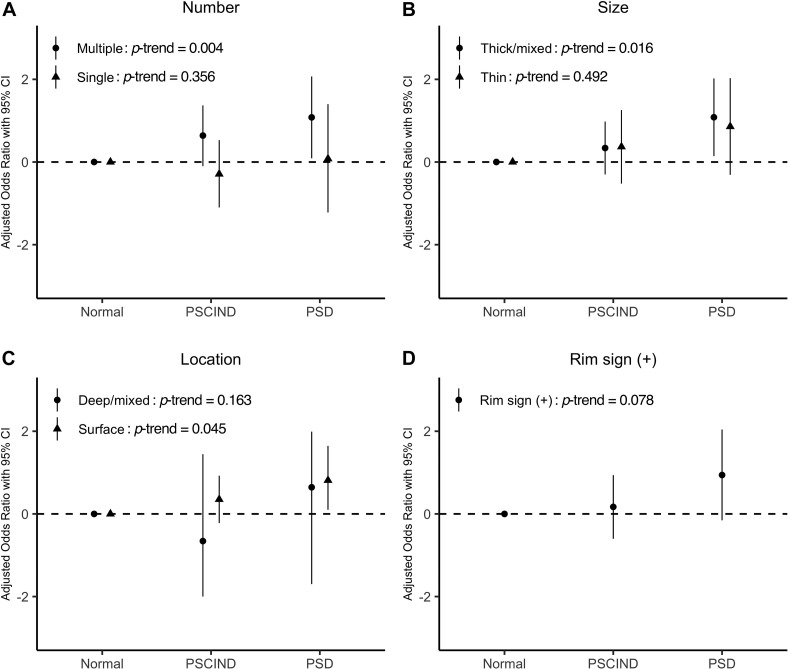
Panel **(A–D)** show the associations between different characteristics of calcified plaques and post-stroke cognitive impairment. Levels of post-stroke cognitive function are shown on the x-axis. Values of odds ratio of specific characteristics of calcified plaques (number, size, location, and rim sign) are transformed using logarithm (log10) and are displayed on the y-axis. Model was adjusted for age, sex, years of education, and baseline NIHSS. *P* for trend is given in each case. NIHSS, National Institute of Health stroke scale; PSCIND, post-stroke cognitive impairment non-dementia; PSD, post-stroke dementia.

Table 2 lists the association between carotid plaque characteristic and PSCI. In logistic regression, the presence of calcification on carotid arteries predicted an increased risk of PSD (*OR* = 7.15; 95% CI: 1.07–47.69). Moreover, we found that the presence of multiple, thick/mixed, surface calcifications were significantly associated with PSD, whereas single, thin, deep/mixed calcifications were not (Figure 2). Participants with multiple, thick/mixed, surface calcifications exhibited an increased risk of PSCI compared to those without calcification (*P*_*trend*_ = 0.004, 0.016, 0.045, respectively). Carotid artery stenosis also significantly increased the risk of PSD (*OR* = 6.42; 95% CI: 1.03–40.15). Rim sign, RI and soft plaque density showed no relationship with PSD in any model. In addition, no association was found between any type of carotid plaque or stenosis and PSCIND.

**TABLE 2:** Association between carotid plaque characteristics and post-stroke cognitive impairment.

		**Normal (*N* = 27)**	**PSCIND (*N* = 75)**	**PSD (*N* = 26)**
Calcification	Model 1	1.0 (ref)	2.31 (0.61-8.71)	7.15 (1.07-47.69)*
	Model 2	1.0 (ref)	2.09 (0.45-9.79)	8.72 (1.03-76.99)*
Soft plaque density	Model 1	1.0 (ref)	1.00 (0.96-1.06)	1.03 (0.98-1.09)
	Model 2	1.0 (ref)	1.00 (0.92-1.10)	1.03 (0.93-1.15)
Remodeling index	Model 1	1.0 (ref)	0.35 (0.03-4.06)	1.35 (0.09-19.46)
	Model 2	1.0 (ref)	0.27 (0.02-4.94)	0.83 (0.03-21.48)
Carotid artery stenosis	Model 1	1.0 (ref)	3.34 (0.57-19.46)	6.42 (1.03-40.15)*
	Model 2	1.0 (ref)	5.41 (0.76-38.51)	10.73 (1.34-85.59)*

*P < 0.05. All results are presented as OR with 95%CI. Model 1 was adjusted for age, sex, years of education, and baseline NIHSS; Model 2 was additionally adjusted for hypertension, hyperlipidemia, WMH, and carotid artery stenosis (for calcification, soft plaque density, and remodeling index) based on Model 1.NIHSS, National Institute of Health stroke scale; PSCIND, post-stroke cognitive impairment non-dementia; PSD, post-stroke dementia; WMH, white matter hyperintensity.

**FIGURE 3:**
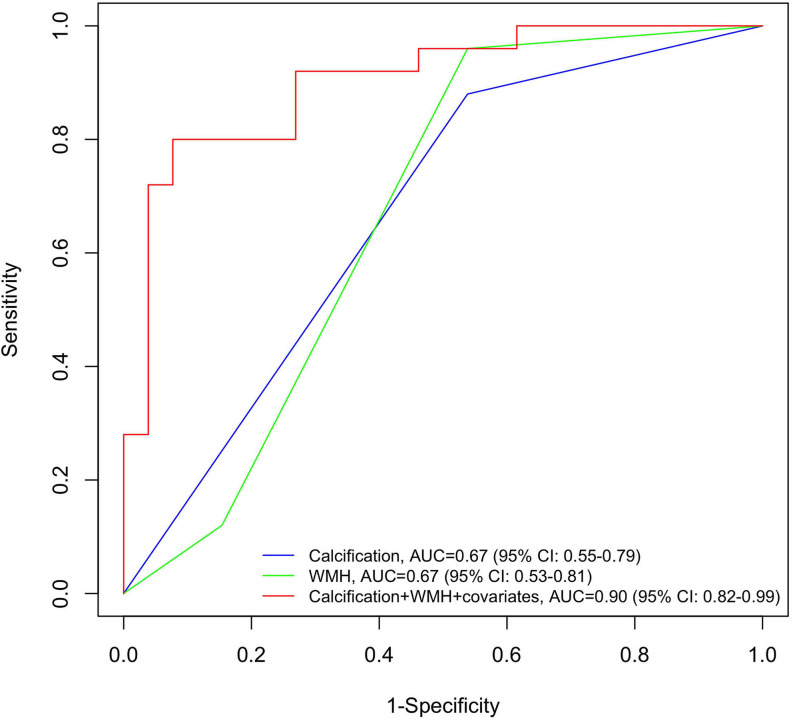
Receiver operating characteristic (ROC) curves for post-stroke dementia by neuroimaging determinants. Blue line: the ROC curve obtained from calcification; green line: the ROC curve obtained from WMH; red line: the ROC curve obtained from the full adjusted model, including calcification, WMH and covariates (age, years of education, and baseline NIHSS). NIHSS, National Institute of Health stroke scale; ROC, receiver operating characteristic; WMH, white matter hyperintensity.

Figure 3 depicts three logistic regression models predicting PSD. The presence of carotid calcification yielded an area under curve (AUC) of 0.67 (95% CI: 0.55–0.79), similar to the result of WMH (AUC = 0.67, 95% CI: 0.53–0.81). When calcification was combined with WMH and other covariates (including age, years of education and baseline NIHSS), the prediction model reached an AUC of 0.90 with 95% CI 0.82 to 0.99.

### Carotid Calcification and Cognitive Impairment in Different Domains

The association between carotid plaque characteristics and cognitive impairment in different domains is displayed in Table 3. In liner regression, most types of calcifications correlated with decreased MMSE score. Moreover, multiple and surface calcifications were found to be associated with decreased *Z* scores in memory domain. We also found that soft plaque density and carotid artery stenosis had a negative impact on cognitive function in execution and visuospatial domain, respectively.

**TABLE 3:** Association between carotid plaque characteristics and post-stroke cognitive impairment in different domains.

		**MMSE**	**Memory**	**Execution**	**Language**	**Visuospatial**
**Calcification**						
Presence	Model 1	−0.66 (0.18)*	−0.34 (0.22)	−0.22 (0.21)	−0.34 (0.23)	−0.41 (0.21)
	Model 2	−0.69 (0.19)*	−0.46 (0.23)*	−0.21 (0.22)	−0.30 (0.24)	−0.38 (0.22)
Number						
Single	Model 1	−0.4 (0.25)	0.03 (0.31)	−0.07 (0.29)	0.06 (0.28)	−0.09 (0.32)
	Model 2	−0.26 (0.3)	0.26 (0.36)	0.20 (0.32)	0.12 (0.32)	0.08 (0.36)
Multiple	Model 1	−0.75 (0.19)*	−0.47 (0.23)*	−0.30 (0.22)	−0.45 (0.25)	−0.43 (0.22)
	Model 2	−0.78 (0.2)*	−0.60 (0.23)*	−0.28 (0.23)	−0.38 (0.26)	−0.39 (0.24)
Size						
Thin	Model 1	−0.76 (0.23)*	−0.19 (0.29)	−0.27 (0.25)	−0.34 (0.26)	−0.34 (0.25)
	Model 2	−0.69 (0.27)*	−0.15 (0.33)	−0.14 (0.29)	−0.38 (0.31)	−0.25 (0.29)
Thick/mixed	Model 1	−0.62 (0.2)*	−0.39 (0.23)	−0.21 (0.23)	−0.29 (0.26)	−0.35 (0.24)
	Model 2	−0.62 (0.21)*	−0.46 (0.24)	−0.17 (0.25)	−0.17 (0.27)	−0.31 (0.26)
Location						
Surface	Model 1	−0.64 (0.18)*	−0.38 (0.23)	−0.22 (0.20)	−0.31 (0.23)	−0.40 (0.21)
	Model 2	−0.67 (0.19)*	−0.48 (0.24)*	−0.22 (0.22)	−0.30 (0.24)	−0.39 (0.22)
Deep/mixed	Model 1	−1.13 (0.39)*	0.27 (0.42)	−0.55 (0.47)	−0.36 (0.37)	0.01 (0.49)
	Model 2	−1.14 (0.43)*	0.45 (0.46)	−0.52 (0.49)	0.11 (0.52)	0.04 (0.54)
Rim sign						
Rim sign (+)	Model 1	−0.61 (0.22)*	−0.28 (0.23)	−0.06 (0.26)	−0.31 (0.25)	−0.19 (0.28)
	Model 2	−0.52 (0.24)*	−0.27 (0.25)	0.06 (0.28)	−0.33 (0.33)	−0.12 (0.31)
**Soft plaque density**	Model 1	−0.01 (0.01)	0.01 (0.01)	−0.01 (0.01)*	0.01 (0.01)	0.01 (0.01)
	Model 2	−0.01 (0.01)	0.01 (0.01)	−0.02 (0.01)*	0.01 (0.01)	0.01 (0.01)
**Remodeling index**	Model 1	−0.07 (0.31)	−0.10 (0.36)	0.43 (0.34)	−0.23 (0.36)	0.39 (0.33)
	Model 2	−0.17 (0.33)	−0.33 (0.37)	0.46 (0.37)	0.14 (0.42)	0.48 (0.35)
**Carotid artery stenosis**	Model 1	−0.05 (0.13)	0.05 (0.15)	−0.16 (0.14)	−0.16 (0.16)	−0.30 (0.14)*
	Model 2	−0.07 (0.13)	0.08 (0.15)	−0.16 (0.14)	−0.16 (0.16)	−0.32 (0.14)*

*P < 0.05. All results are presented as standardized coefficients β with standard error. Values represent differences in Z score for MMSE and cognitive domain. Complete data of MMSE and each cognitive domain was available in 123 participants. Model 1 was adjusted for age, sex, years of education, and baseline NIHSS; Model 2 was additionally adjusted for hypertension, hyperlipidemia, WMH, and carotid artery stenosis (for calcification, soft plaque density, and remodeling index) based on Model 1.MMSE, Mini-mental State Examination; NIHSS, National Institute of Health stroke scale; WMH, white matter hyperintensity.

## Discussion

This study investigated the association between carotid atherosclerotic calcification and PSCI in hospitalized patients with stroke. We found the presence of calcification, especially multiple, thick/mixed, and surface calcification on carotid arteries to be associated with PSCI, after full adjustment (demographics, vascular risk factors, CSVD and stroke severity). In general, our findings suggest that the quantity and location of calcification in carotid atherosclerotic plaques may be independent indicators for PSCI.

Atherosclerosis is a chronic disease affecting the structure and function of blood vessels, leading to neurovascular dysfunction which causes cognitive decline (Shabir et al., 2018). The progressive formation of plaque and increased stiffness in carotid arteries accelerates cognitive dysfunction, particularly vascular cognitive impairment, by decreasing cerebral blood flow and promoting a breakdown of neurovascular coupling (Girouard and Iadecola, 2006; Jefferson et al., 2018). Atherosclerotic calcification represents an advanced stage of atherosclerosis pathogenesis. Although few studies have investigated whether atherosclerotic calcification is related with PSCI, the association between calcification and cognitive impairment at the pre-clinical stage has been identified by a range of research. In Rotterdam study, larger calcification volume was found to be associated with a higher risk of dementia and cognitive decline in a population-based cohort (Bos et al., 2012, 2015). In the CARDIA study, subclinical atherosclerotic calcification was related with poorer psychomotor speed and memory in midlife in a community-based sample (Reis et al., 2013). Another hospital-based study proved that common carotid artery calcification increased the risk of cognitive impairment and dementia, and this association appeared independent of arterial stiffness (Di Daniele et al., 2019). All of these findings supported the hypothesis that the presence and amount of calcification is related to cognitive impairment, which is supported by our results.

The association between calcified atherosclerosis and PSCI could be explained by several potential pathways. Firstly, chronic hypoperfusion may enhance this association. Carotid artery stiffness caused by calcification may attenuate resting cerebral blood flow in several regions and increase blood-brain barrier permeability, which can lead to disruption of microcirculation and vasculature integrity (Muhire et al., 2019). This may contribute to the pathogenesis of vascular dementia, and in turn enhance comorbidity with neurodegenerative diseases (Alzheimer’s disease) (Ueno et al., 2016). Secondly, carotid atherosclerotic calcification could increase the risk and severity of stroke, subsequently resulting in a higher likelihood of PSCI. However, our findings showed that the significant association between calcification and PSCI still existed after adjusting baseline NIHSS score. This suggested that stroke played only a limited role in the progression of PSCI, although it cannot be entirely excluded as a contributing factor. Finally, multiple microemboli induced by unstable plaques may accelerate cognitive impairment after stroke. In our study, we found that patients with multiple, thick and surface calcifications were more likely to develop to PSCI. The location, shape, size and gap between calcifications change mechanical stresses and affect cap stability (Cardoso et al., 2014). Compared with deep or mixed calcifications, surface calcifications increase the risk of neovessel rupture and thrombosis by elevating plaque surface stress (Li et al., 2007; Alfonso et al., 2013). Similar mechanisms apply to multiple calcified plaques (Kelly-Arnold et al., 2013). Thus, these calcifications may be representative of vulnerable plaques. This notion is supported by findings, that superficial, multiple and thick (≥2 mm) calcifications were independently related to the presence of intra plaque hemorrhage (Eisenmenger et al., 2016; Lin et al., 2017; Yang et al., 2018). Vulnerable plaques may produce both clinically evident emboli and subclinical multiple microemboli leading to brain atrophy and silent brain infarcts, thus proceeding cognitive impairment (Dempsey et al., 2010).

Moreover, we found that carotid calcification, artery stenosis and soft plaque density each had different effects on performance in each cognitive domain. In agreement with others studies, carotid artery stenosis and soft plaque density were significantly associated with impaired execution and visuospatial function (Bos et al., 2012; Gong et al., 2020). These associations could be explained by the disruption of anterior and posterior watershed areas caused by hypoperfusion. Whereas, multiple and surface calcifications were found to predominantly disrupt function in the memory domain. These unstable carotid atherosclerotic plaques tend to be more likely to induce multiple microemboli, and cause strategic infarcts within the cortex (Hase et al., 2019). Unlike hypoperfusion, emboli may cause more diffuse damage to cortical areas and subsequently impair memory (Fearn et al., 2001). The multiple cognitive domains involved in associations between carotid atherosclerosis and PSCI revealed the sophisticated underlying mechanisms, which need to be further assessed and confirmed through pathological evidence.

The strengths of our study include its longitudinal study design, reliable brain imaging acquisition method and the detailed cognitive function questionnaire including different domains. Several potential limitations should also be illustrated. Firstly, in our study, atherosclerotic calcification and plaque characteristics were recognized by brain/neck CTA. CTA is a sensitive, observer-independent, and reliable tool to evaluate calcification. Compared with high-resolution MRI, CTA is faster, with fewer contraindications, and is generally more feasible. It is therefore widely used in clinical practice, especially for stroke patients. However, CTA has less capacity to identify soft plaque components, such as ulceration and intra plaque hemorrhage. It is also difficult to determine the border of calcification due to blooming artifacts at the vessel wall, which may cause bias in patients with severe calcification. Secondly, the associations which we have found, may be confounded by the lack of quantitative analysis of calcification. Although we categorized calcifications based on their size and numbers, the overall burden of calcification may still influence the result. Thirdly, the limited cases with PSD in this study cause quite broad confidence intervals, especially when calcification was treated as a binary covariate. As shown in Table 1, the distribution of calcification showed significant difference between normal, PSCIND, and PSD groups using univariate analysis. A larger sample size is supposed to make our conclusion more accurate and solid. Finally, selection bias should also be considered when explaining these associations. In our study design, patients who could not complete cognitive function assessment due to hemiplegia or aphasia at baseline were excluded. Thus, the included subjects in our trial had relatively lower NIHSS score (4.0 points) than average. This selection bias may lead to an underestimation of the effect of stroke on PSCI. Additional screening utilizing a cognitive function scale which is more suitable for stroke patients, especially those whom cannot speak or write, is needed in future studies.

## Conclusion

In this hospital-based study, the presence of carotid calcification was found to be associated with PSCI. This association depended on the size, number, and location of calcifications. In addition to CSVD, large artery atherosclerosis should be considered as an important risk factor for PSD.

## Data Availability Statement

The data used in this study are available upon reasonable request from the corresponding author.

## Ethics Statement

The studies involving human participants were reviewed and approved by the Ethics Committee of the Fudan University, Shanghai, China. The patients/participants provided their written informed consent to participate in this study.

## Author Contributions

YinW, MD, QD, and MC conceived the cohort and take responsibility for its all aspects. YinW and CL wrote the manuscript. YinW completed all the statistical analysis supported by YY and MC. MD led the data collection supported by YizW, PL, and MC. CL, LL, and YY reviewed and commented on the data analysis and drafts. All authors interpreted data, contributed to critical revisions, and approved the final version of the article.

## Conflict of Interest

The authors declare that the research was conducted in the absence of any commercial or financial relationships that could be construed as a potential conflict of interest.

## Abbreviations

AUC: area under curve
CI: confidence interval
CSVD: cerebral small vessel disease
CTA: computer tomography angiography
ICAS: intracranial artery stenosis
IQR: interquartile range
MMSE: Mini − mental State Examination
MRI: magnetic resonance imaging
NIHSS: National Institute of Health stroke scale
OR: odds ratio
PSCI: post-stroke cognitive impairment
PSCIND: post-stroke cognitive impairment with no dementia
PSD: post-stroke dementia
RI: remodeling index
WMH: white matter hyperintensity.

## References

[B1] Alfonso F. Gonzalo N. Nunez-Gil I. Banuelos C. (2013). Coronary thrombosis from large, nonprotruding, superficial calcified coronary plaques. *J. Am. Coll. Cardiol.* 62:2254. 10.1016/j.jacc.2013.04.106 24080108

[B2] Arba F. Quinn T. J. Hankey G. J. Lees K. R. Wardlaw J. M. Ali M. (2018). Enlarged perivascular spaces and cognitive impairment after stroke and transient ischemic attack. *Int. J. Stroke* 13 47–56. 10.1177/1747493016666091 27543501

[B3] Bos D. Vernooij M. W. de Bruijn R. F. Koudstaal P. J. Hofman A. Franco O. H. (2015). Atherosclerotic calcification is related to a higher risk of dementia and cognitive decline. *Alzheimers Dement.* 11 639–647.e1. 10.1016/j.jalz.2014.05.1758 25150731

[B4] Bos D. Vernooij M. W. Elias-Smale S. E. Verhaaren B. F. Vrooman H. A. Hofman A. (2012). Atherosclerotic calcification relates to cognitive function and to brain changes on magnetic resonance imaging. *Alzheimers Dement.* 8(5 Suppl) S104–S111. 10.1016/j.jalz.2012.01.008 22537801

[B5] Cardoso L. Kelly-Arnold A. Maldonado N. Laudier D. Weinbaum S. (2014). Effect of tissue properties, shape and orientation of microcalcifications on vulnerable cap stability using different hyperelastic constitutive models. *J. Biomech.* 47 870–877. 10.1016/j.jbiomech.2014.01.010 24503048 PMC4019736

[B6] Chaudhari T. S. Verma R. Garg R. K. Singh M. K. Malhotra H. S. Sharma P. K. (2014). Clinico-radiological predictors of vascular cognitive impairment (VCI) in patients with stroke: a prospective observational study. *J. Neurol. Sci.* 340 150–158. 10.1016/j.jns.2014.03.018 24680559

[B7] Committee (1991). North American symptomatic carotid endarterectomy trial. Methods, patient characteristics, and progress. *Stroke* 22 711–720. 10.1161/01.str.22.6.711 2057968

[B8] Dempsey R. J. Vemuganti R. Varghese T. Hermann B. P. (2010). A review of carotid atherosclerosis and vascular cognitive decline: a new understanding of the keys to symptomology. *Neurosurgery* 67 484–494. 10.1227/01.NEU.0000371730.11404.36 20644437 PMC2908960

[B9] Di Daniele N. Celotto R. Alunni Fegatelli D. Gabriele M. Rovella V. Scuteri A. (2019). Common carotid artery calcification impacts on cognitive function in older patients. *High Blood Press. Cardiovasc. Prev.* 26 127–134. 10.1007/s40292-019-00301-z 30779026

[B10] Eisenmenger L. B. Aldred B. W. Kim S. E. Stoddard G. J. de Havenon A. Treiman G. S. (2016). Prediction of carotid intraplaque hemorrhage using adventitial calcification and plaque thickness on CTA. *AJNR Am. J. Neuroradiol.* 37 1496–1503. 10.3174/ajnr.A4765 27102316 PMC7960279

[B11] Fearn S. J. Pole R. Wesnes K. Faragher E. B. Hooper T. L. McCollum C. N. (2001). Cerebral injury during cardiopulmonary bypass: emboli impair memory. *J. Thorac. Cardiovasc. Surg.* 121 1150–1160. 10.1067/mtc.2001.114099 11385383

[B12] Girouard H. Iadecola C. (2006). Neurovascular coupling in the normal brain and in hypertension, stroke, and Alzheimer disease. *J. Appl. Physiol.* 100 328–335. 10.1152/japplphysiol.00966.2005 16357086

[B13] Gong L. Wang H. Dong Q. Zhu X. Zheng X. Gu Y. (2020). Intracranial atherosclerotic stenosis is related to post-stroke cognitive impairment: a cross-sectional study of minor stroke. *Curr. Alzheimer Res.* 17 177–184. 10.2174/1567205017666200303141920 32124696

[B14] Gottesman R. F. Hillis A. E. (2010). Predictors and assessment of cognitive dysfunction resulting from ischaemic stroke. *Lancet Neurol.* 9 895–905. 10.1016/s1474-4422(10)70164-2 20723846 PMC3592203

[B15] Gregoire S. M. Smith K. Jager H. R. Benjamin M. Kallis C. Brown M. M. (2012). Cerebral microbleeds and long-term cognitive outcome: longitudinal cohort study of stroke clinic patients. *Cerebrovasc. Dis.* 33 430–435. 10.1159/000336237 22456577

[B16] Hase Y. Polvikoski T. M. Ihara M. Hase M. Zafar R. Stevenson W. (2019). Carotid artery disease in post-stroke survivors and effects of enriched environment on stroke pathology in a mouse model of carotid artery stenosis. *Neuropathol. Appl. Neurobiol.* 45 681–697. 10.1111/nan.12550 30947376

[B17] Jacquin A. Binquet C. Rouaud O. Graule-Petot A. Daubail B. Osseby G. V. (2014). Post-stroke cognitive impairment: high prevalence and determining factors in a cohort of mild stroke. *J. Alzheimers Dis.* 40 1029–1038. 10.3233/JAD-131580 24577459

[B18] Jefferson A. L. Cambronero F. E. Liu D. Moore E. E. Neal J. E. Terry J. G. (2018). Higher aortic stiffness is related to lower cerebral blood flow and preserved cerebrovascular reactivity in older adults. *Circulation* 138 1951–1962. 10.1161/CIRCULATIONAHA.118.032410 30018169 PMC6394409

[B19] Kandiah N. Chander R. J. Lin X. Ng A. Poh Y. Y. Cheong C. Y. (2016). Cognitive impairment after mild stroke: development and validation of the SIGNAL2 risk score. *J. Alzheimers Dis.* 49 1169–1177. 10.3233/JAD-150736 26599056

[B20] Kebets V. Gregoire S. M. Charidimou A. Barnes J. Rantell K. Brown M. M. (2015). Prevalence and cognitive impact of medial temporal atrophy in a hospital stroke service: retrospective cohort study. *Int. J. Stroke* 10 861–867. 10.1111/ijs.12544 26043795

[B21] Kelly-Arnold A. Maldonado N. Laudier D. Aikawa E. Cardoso L. Weinbaum S. (2013). Revised microcalcification hypothesis for fibrous cap rupture in human coronary arteries. *Proc. Natl. Acad. Sci. U.S.A.* 110 10741–10746. 23733926 10.1073/pnas.1308814110 PMC3696743

[B22] Leys D. Hénon H. Mackowiak-Cordoliani M.-A. Pasquier F. (2005). Poststroke dementia. *Lancet Neurol.* 4 752–759. 10.1016/s1474-4422(05)70221-0 16239182

[B23] Li S. Fang F. Cui M. Jiang Y. Wang Y. Kong X. (2019). Incidental findings on brain MRI among Chinese at the age of 55-65 years: the Taizhou imaging study. *Sci. Rep.* 9:464. 10.1038/s41598-018-36893-0 30679548 PMC6345793

[B24] Li X. Ma X. Lin J. He X. Tian F. Kong D. (2017). Severe carotid artery stenosis evaluated by ultrasound is associated with post stroke vascular cognitive impairment. *Brain Behav.* 7:e00606. 10.1002/brb3.606 28127524 PMC5256189

[B25] Li Z. Y. Howarth S. Tang T. Graves M. U-King-Im J. Gillard J. H. (2007). Does calcium deposition play a role in the stability of atheroma? Location may be the key. *Cerebrovasc. Dis.* 24 452–459. 10.1159/000108436 17878727

[B26] Lin R. Chen S. Liu G. Xue Y. Zhao X. (2017). Association between carotid atherosclerotic plaque calcification and intraplaque hemorrhage: a magnetic resonance imaging study. *Arterioscler. Thromb. Vasc. Biol.* 37 1228–1233. 10.1161/ATVBAHA.116.308360 28450297

[B27] Lu D. Ren S. Zhang J. Sun D. (2016). Vascular risk factors aggravate cognitive impairment in first-ever young ischaemic stroke patients. *Eur. J. Neurol.* 23 940–947. 10.1111/ene.12967 26917058

[B28] Miura T. Matsukawa N. Sakurai K. Katano H. Ueki Y. Okita K. (2011). Plaque vulnerability in internal carotid arteries with positive remodeling. *Cerebrovasc. Dis. Extra* 1 54–65. 10.1159/000328645 22566983 PMC3343763

[B29] Molad J. Kliper E. Korczyn A. D. Ben Assayag E. Ben Bashat D. Shenhar-Tsarfaty S. (2017). Only white matter hyperintensities predicts post-stroke cognitive performances among cerebral small vessel disease markers: results from the TABASCO study. *J. Alzheimers Dis.* 56 1293–1299. 10.3233/JAD-160939 28157096

[B30] Muhire G. Iulita M. F. Vallerand D. Youwakim J. Gratuze M. Petry F. R. (2019). Arterial stiffness due to carotid calcification disrupts cerebral blood flow regulation and leads to cognitive deficits. *J. Am. Heart Assoc.* 8:e011630. 10.1161/JAHA.118.011630 31057061 PMC6512142

[B31] Rasquin S. M. Verhey F. R. van Oostenbrugge R. J. Lousberg R. Lodder J. (2004). Demographic and CT scan features related to cognitive impairment in the first year after stroke. *J. Neurol. Neurosurg. Psychiatry* 75 1562–1567. 10.1136/jnnp.2003.024190 15489388 PMC1738816

[B32] Reis J. P. Launer L. J. Terry J. G. Loria C. M. Zeki Al Hazzouri A. Sidney S. (2013). Subclinical atherosclerotic calcification and cognitive functioning in middle-aged adults: the CARDIA study. *Atherosclerosis* 231 72–77. 10.1016/j.atherosclerosis.2013.08.038 24125414 PMC3828555

[B33] Samuels O. B. Joseph G. J. Lynn M. J. Smith H. A. Chimowitz M. I. (2000). A standardized method for measuring intracranial arterial stenosis. *AJNR Am. J. Neuroradiol.* 21 643–646. 10782772 PMC7976653

[B34] Shabir O. Berwick J. Francis S. E. (2018). Neurovascular dysfunction in vascular dementia, Alzheimer’s and atherosclerosis. *BMC Neurosci.* 19:62. 10.1186/s12868-018-0465-5 30333009 PMC6192291

[B35] Sun J. H. Tan L. Yu J. T. (2014). Post-stroke cognitive impairment: epidemiology, mechanisms and management. *Ann. Transl. Med.* 2:80. 10.3978/j.issn.2305-5839.2014.08.05 25333055 PMC4200648

[B36] Ueno M. Chiba Y. Matsumoto K. Murakami R. Fujihara R. Kawauchi M. (2016). Blood-brain barrier damage in vascular dementia. *Neuropathology* 36 115–124. 10.1111/neup.12262 26607405

[B37] U-King-Im J. M. Fox A. J. Aviv R. I. Howard P. Yeung R. Moody A. R. (2010). Characterization of carotid plaque hemorrhage: a CT angiography and MR intraplaque hemorrhage study. *Stroke* 41 1623–1629. 10.1161/STROKEAHA.110.579474 20576955

[B38] Wardlaw J. M. Smith E. E. Biessels G. J. Cordonnier C. Fazekas F. Frayne R. (2013). Neuroimaging standards for research into small vessel disease and its contribution to ageing and neurodegeneration. *Lancet Neurol.* 12 822–838. 10.1016/s1474-4422(13)70124-8 23867200 PMC3714437

[B39] Wen H. M. Mok V. C. T. Fan Y. H. Lam W. W. M. Tang W. K. Wong A. (2004). Effect of white matter changes on cognitive impairment in patients with lacunar infarcts. *Stroke* 35 1826–1830. 10.1161/01.Str.0000133686.29320.58 15205490

[B40] Yang J. Pan X. Zhang B. Yan Y. Huang Y. Woolf A. K. (2018). Superficial and multiple calcifications and ulceration associate with intraplaque hemorrhage in the carotid atherosclerotic plaque. *Eur. Radiol.* 28 4968–4977. 10.1007/s00330-018-5535-7 29876705 PMC6223859

