# Supplementary material for: Carotid Atherosclerotic Calcification Characteristics Relate to Post-stroke Cognitive Impairment
Source: Front Aging Neurosci. 2021 May 25;13:682908. doi: 10.3389/fnagi.2021.682908 (PMC8185032; doi:10.3389/fnagi.2021.682908)
Supplement: Supplementary file 1 [file Data_Sheet_1.PDF]

SUPPLEMENTAL MATERIAL

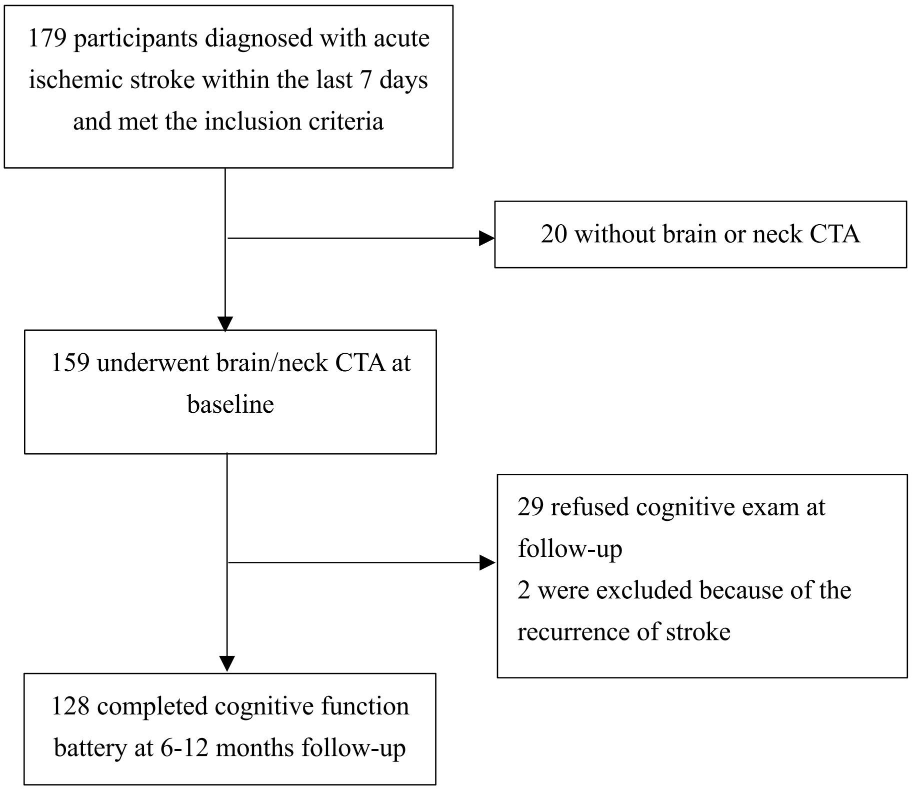

Supplementary Figure I. Flowchart of the inclusion process of the study sample.

Supplementary Table I. Baseline profiles across participants involved in the study and lost in follow-up.

	Participants involved in analysis (N=128)	Participants lost in follow-up (N=29)	*P* value
**Demographic characteristics**			
Age, mean ± SD	62.1 ± 12.2	60.5 ± 13.9	0.54
Female, n (%)	37 (28.9)	8 (27.6)	0.89
Education year#, median (IQR)	12.0 (9.0, 15.0)	12.0 (9.0, 12.0)	0.43
Smoking (ever), n (%)	65 (50.8)	19 (65.5)	0.15
Alcohol consumption (ever), n (%)	42 (32.8)	8 (27.6)	0.59
Hypertension, n (%)	93(72.7)	22 (75.9)	0.73
Diabetes mellitus, n (%)	38 (29.7)	12 (41.4)	0.22
Hyperlipidemia, n (%)	51 (39.8)	16 (55.2)	0.13
**Baseline stroke severity**			
NIHSS at baseline, median (IQR)	4.0 (2.0, 6.0)	4.0 (2.0, 8.0)	0.30
**Cerebral small vessel diseases**			
Lacune, n (%)	53 (41.4)	11 (37.9)	0.85
WMH			0.34
Mild, n (%)	27 (21.1)	6 (20.7)	
Moderate, n (%)	80 (62.5)	18 (62.1)	
Severe, n (%)	21 (16.4)	5 (17.2)	
**Atherosclerotic characteristics**			
Calcification, n (%)	96 (75)	24 (82.8)	0.37
Soft plaque density (Hu)#, mean ± SD	40.0 ± 24.1	40.8 ± 15.2	0.93
Remodeling index, median (IQR)	1.0 (1.0, 1.2)	1.0 (1.0, 1.0)	0.12
Carotid artery stenosis			0.44
Mild, n (%)	107 (83.6)	26 (89.7)	
Moderate or severe, n (%)	21 (16.4)	3 (10.3)	

#3 subjects had missing data for years of education and 18 for soft plaque density due to small plaque volume.

Abbreviations: IQR = interquartile range; NIHSS = National Institute of Health stroke scale; WMH = white matter hyperintensity.
